# Opposing Natural Killer and Macrophage Associations Are Masked Within a CD16/IL-15/IL-15RA Composite Score in Metastatic Urothelial Carcinoma

**DOI:** 10.3390/ijms27208943

**Published:** 2026-10-09

**Authors:** Erkut Demirciler, Faruk Recep Özalp

**Affiliations:** 1 Department of Medical Oncology, Izmir City Hospital, Izmir 35540, Turkey; 2 Department of Medical Oncology, Osmaniye Education and Research Hospital, Osmaniye 80000, Turkey; ozalpfarukrecep@gmail.com

**Keywords:** urinary bladder neoplasms, antibody-dependent cell cytotoxicity, natural killer cells, interleukin-15

## Abstract

Programmed death-ligand 1 (PD-L1) poorly predicts immunotherapy benefit in urothelial carcinoma. Avelumab retains an Fc region capable of antibody-dependent cellular cytotoxicity (ADCC), prompting a proposed composite biomarker of CD16, interleukin-15 (IL-15), and IL-15RA. We assessed its construct validity. We built a transcriptomic surrogate in IMvigor210 (348 patients with metastatic urothelial carcinoma treated with atezolizumab, an effector-silenced anti-PD-L1 antibody) and analyzed overall survival with cohort-stratified Cox regression, decomposing the composite against natural killer (NK) cell and macrophage signatures. Among 232 deaths, the composite was not associated with survival (HR 1.03, *p* = 0.64), whereas tumor mutational burden retained its expected association (HR 0.75). Analyzed separately, the NK signal was protective (HR 0.83), and the macrophage signal was uninformative (HR 1.04); modeled jointly, both strengthened in opposite directions (NK HR 0.71, *p* = 0.0001; macrophage HR 1.29, *p* = 0.002). Removing CD16, but not either IL-15 component, reduced the composite’s correlation with macrophage content, and MCP-counter deconvolution reproduced the opposing associations. The composite reads null because its compartments carry opposing associations that mask one another, not because it is specific for ADCC. CD16 should be replaced with NK-restricted markers, and ADCC-competency scores must add value beyond NK abundance.

## 1. Introduction

Bladder cancer is the tenth most commonly diagnosed cancer worldwide, with approximately 573,000 new cases and 212,000 deaths estimated in 2020 [1]. Platinum-based chemotherapy was long the standard first-line treatment for advanced urothelial carcinoma, and avelumab, an anti-programmed death-ligand 1 (PD-L1) antibody, is indicated as first-line maintenance therapy in patients whose disease has not progressed on platinum [2,3]. Among patients whose disease has progressed after a previous platinum-based strategy, pembrolizumab or atezolizumab is a recommended option. In contrast, enfortumab vedotin plus pembrolizumab has more recently been approved in the upfront setting [4]. Since that approval, the population in which platinum-based chemotherapy followed by avelumab maintenance is used has narrowed. However, this pathway remains relevant for patients ineligible for or progressing after that combination.

Over the past decade, many studies have evaluated prognostic and predictive biomarkers for immune checkpoint blockade in advanced urothelial carcinoma, including PD-L1 expression [5], tumor mutational burden [6], alterations in DNA damage response and repair genes [7], and gene expression signatures [8]. Despite these efforts, no biomarker has been consistently translated into routine clinical practice.

For avelumab specifically, the difficulty with PD-L1 is more fundamental than imperfect discrimination, and it takes two forms. The first is a measurement problem: PD-L1 positivity in JAVELIN Bladder 100 was defined using the Ventana SP263 assay, originally developed for durvalumab, whereas the avelumab development program used the Dako PD-L1 IHC 73-10 assay [2,9,10]. These assays are not interchangeable, and the same tumor may be classified as positive or negative depending on the reagent used [11].

The second is a clinical problem that persists even when the assay is held constant. In JAVELIN Bladder 100, no significant interaction between treatment and PD-L1 status was observed for overall survival, and avelumab is accordingly approved without regard to PD-L1 status [2,12]. Within the control arm of that trial, median overall survival was longer in the PD-L1-positive population than overall, indicating that PD-L1 expression in this setting carries a prognostic component rather than functioning purely as a predictor of benefit [2].

Tumor PD-L1 expression therefore does not identify the patients who benefit from avelumab. If the target antigen does not identify who benefits, the composition of the immune infiltrate, and of the innate compartment in particular, merits evaluation.

Avelumab differs mechanistically from other anti-PD-L1 antibodies in a way that makes this question specific to it. Atezolizumab and durvalumab carry engineered Fc regions in which effector function has been abolished. In contrast, avelumab retains a functional IgG1 Fc that can engage FcγRIIIa (CD16) on natural killer (NK) cells and trigger antibody-dependent cellular cytotoxicity (ADCC) against PD-L1-expressing tumor cells in vitro [13,14]. Exploratory analyses of JAVELIN Bladder 100 reported that survival benefit was associated with the number of alleles encoding high-affinity variants of activating Fcγ receptors [15]. These analyses were exploratory, but FcγR genotype has been associated with the efficacy of other therapeutic IgG1 antibodies, including rituximab in follicular lymphoma [16] and trastuzumab in breast cancer [17], supporting the biological plausibility of an ADCC contribution.

We therefore hypothesized that a tumor microenvironment with abundant CD16-positive effectors, supported by interleukin-15 (IL-15) and IL-15 receptor alpha (IL-15RA), the obligate cytokine axis for NK survival, priming, and cytotoxicity [18,19], would derive disproportionate benefit from avelumab. We proposed a composite ADCC-competency score combining these three markers for immunohistochemical assessment.

The biological relationships among CD16, the interleukin-15 axis, natural killer cell activity, and ADCC are well established, and we assembled the composite score from that existing framework rather than proposing new biology. The contribution of the present work is analytical: we show that the biological plausibility of the individual components does not establish the construct validity of the composite they form, and that an additive score combining markers expressed by functionally opposed cell populations can read null regardless of how much biology is present.

Before evaluating such a score where its mechanism is known, its construct validity must be established: it must be shown to measure the effector population it is intended to measure. A cohort treated with an effector-silenced antibody permits this while simultaneously testing whether the score behaves as a mechanism-specific marker or merely as a generic index of immune infiltration. Herein, we conducted a retrospective analysis to assess the construct validity of this composite score in patients with advanced urothelial carcinoma treated with atezolizumab in the IMvigor210 phase II clinical trial [5,8,20], a setting in which ADCC cannot occur. We emphasize that this design is necessary but not sufficient: a score that is not inert in an effector-silenced cohort cannot be specific for ADCC, whereas a score that is inert is not thereby validated.

## 2. Results

### 2.1. Patient Population

Baseline characteristics appear in Table 1. Of the 348 patients, 76 were platinum-naive and 272 platinum-pretreated. Median follow-up by reverse Kaplan-Meier was 20.6 months (95% confidence interval [CI] 20.0–21.1), with 232 deaths. Figure 1 summarizes the analytical workflow.

Tumor mutational burden was available for 272 patients, comprising 70 of 76 in cohort 1 and 202 of 272 in cohort 2. Availability was therefore not confined to one cohort, though missingness was more common in the platinum-pretreated group (26% versus 8%). Immune phenotype was available for 284 patients and PD-L1 for 347. We recorded baseline ECOG performance status for all 348 patients and metastatic disease status for 316. The public release does not include age at diagnosis, prior lines of therapy, or hemoglobin concentration.

The two cohorts differed as expected for their clinical definitions. Median overall survival was 14.1 months (95% CI 7.7 to not reached) in the platinum-naive cohort and 8.0 months (95% CI 6.7–10.3) in the platinum-pretreated cohort, confirming the cohort variable’s orientation. Performance status differed markedly: 22.4% of platinum-naive patients had an ECOG score of 2, compared with 0.4% of platinum-pretreated patients, consistent with poor performance status being a common reason for cisplatin ineligibility. Upper tract tumors were also more frequent in the platinum-naive cohort (renal pelvis 29.7% versus 16.9%). These differences reinforce the decision to stratify all models by cohort.

### 2.2. Outcomes of the Composite Score

The score was evenly distributed across the cohort, with 96, 76, 82, and 94 patients with scores of 0, 1, 2, and 3. Overall survival did not differ across the four groups and showed no ordering: median survival was 9.23, 8.08, 7.85, and 9.76 months, respectively, with confidence intervals that overlap almost completely (Table 2, Figure S1).

When modeled continuously, the score had a hazard ratio (HR) of 1.03 (95% CI 0.92–1.15, *p* = 0.64) and a concordance index of 0.510, indistinguishable from chance. Restricting to the 284 patients with complete immune covariate data gave a near-identical estimate (HR 1.02, 95% CI 0.90–1.16, *p* = 0.73). The proportional hazards assumption held (Schoenfeld χ^2^ = 0.43, 1 degree of freedom, *p* = 0.51) and the survival curves did not cross.

With 232 events, the confidence interval excludes per-unit hazard ratios below 0.92 or above 1.15. The analysis therefore rules out moderate associations in either direction, though not small ones.

Analyzed as a continuous variable rather than a dichotomized sum, the composite gave a near-identical result (HR 0.97, 95% CI 0.83–1.12, *p* = 0.65, c = 0.505), indicating that the null does not arise from dichotomization at the median.

Established predictors analyzed by the same pipeline recovered their expected associations in positive-control analyses where real signal exists (Table S1).

Tumor mutational burden was strongly protective (HR 0.75, 95% CI 0.65–0.87, *p* = 0.0001) and gave the highest discrimination of any biomarker we examined (c = 0.588). PD-L1 on immune cells was protective (HR 0.75, 95% CI 0.63–0.88, *p* = 0.0004), as was tumor immune phenotype, though less strongly (HR 0.81, 95% CI 0.67–0.99, *p* = 0.037). PD-L1 on tumor cells showed no association (HR 1.01, 95% CI 0.85–1.20, *p* = 0.92).

### 2.3. Cellular Composition of the Composite Score

To determine which component contributed most to the composite’s association with myeloid content, each component was removed in turn. Removing CD16 reduced the correlation between the composite and the macrophage signature from 0.80 to 0.69 and increased its correlation with NK-restricted markers from 0.50 to 0.55. Removing IL15 or IL15RA did not reduce the macrophage correlation (0.82 and 0.81, respectively). CD16 therefore contributes more myeloid signal to the composite than either interleukin-15 component, consistent with FCGR3A expression by macrophages and neutrophils in addition to NK cells. No single-component removal recovered prognostic information (hazard ratios 0.95 to 0.98; all *p* > 0.45; concordance indices 0.505 to 0.509), indicating that the null association does not arise from one uninformative marker.

The NK and macrophage signatures correlated at rho 0.646, and variance inflation factors in the joint model were 1.58, below conventional thresholds for problematic collinearity. Both coefficients moved further from the null with smaller *p*-values when modeled jointly, consistent with removing mutual confounding rather than collinearity-driven instability.

### 2.4. Outcomes of Composite Score in Immune Infiltration

When cross-tabulated with the trial’s immune phenotype classification, the score showed a strong monotonic relationship (Table 3). Desert tumors fell from 49.4% of patients at score 0 to 7.7% at score 3, while inflamed tumors rose from 6.3% to 57.7%. The score correlated with immune phenotype (Spearman rho 0.50). We therefore extracted genes and constructed the score as intended. The null association reflects what the score comprises, not a failure to measure it.

None of the three constituent genes was associated with overall survival when assessed alone, and all three had concordance indices at or below 0.51 (Table S2).

Among the 284 patients with complete data, the score alone showed an HR of 1.02 (*p* = 0.73). Adding immune phenotype and PD-L1 on immune cells produced a nominally significant association opposite to our hypothesis (HR 1.18, 95% CI 1.02–1.37, *p* = 0.028; Table S3). Because the same patients yielded a null estimate without adjustment, this cannot be attributed to sample loss. In the adjusted model, PD-L1 on immune cells retained its protective association (HR 0.73, *p* = 0.010), whereas immune phenotype did not (HR 0.87, *p* = 0.27).

The score correlates with immune phenotype (0.50) and with PD-L1 on immune cells (0.44). We do not interpret the adjusted coefficient causally.

### 2.5. Outcomes of NK and Macrophage Gene Signatures

Analyzed separately, the NK signature was protective (HR 0.83, 95% CI 0.73–0.95, *p* = 0.0071) while the macrophage signature appeared uninformative (HR 1.04, 95% CI 0.91–1.19, *p* = 0.56).

Modeled together, both associations strengthened substantially and separated in opposite directions: NK HR 0.71 (95% CI 0.60–0.84, *p* = 0.0001) and macrophage HR 1.29 (95% CI 1.09–1.52, *p* = 0.0024), with concordance rising to 0.575 (Table 4). The two signatures correlate at rho 0.65, and each masks the other until both are in the model (Figure 2).

Because the initial NK marker set contains genes shared with CD8-positive T cells, we repeated the analysis using NCR1 and KLRF1 alone. The protective association held (HR 0.77, 95% CI 0.67–0.90, *p* = 0.001, c = 0.572), suggesting the effect is driven by NK cells rather than cytotoxic lymphocytes in general. In that model, the adverse macrophage association attenuated to marginal significance (HR 1.15, 95% CI 1.00–1.32, *p* = 0.056), indicating that the myeloid effect resolves more clearly against total cytotoxic lymphocyte content than against NK abundance alone.

The composite therefore sums a protective and an adverse compartment. The null result therefore reflects mutual masking of opposing associations within a summed score, not absence of biology.

The composite correlated more strongly with the macrophage signature (rho 0.75) than with NK-restricted markers (rho 0.47).

Among its components, the IL-15 axis showed the strongest correlation with the composite (rho 0.88) but carried no prognostic information (HR 0.99, 95% CI 0.87–1.12, *p* = 0.86, c = 0.503). That axis also correlated more closely with the macrophage signature (rho 0.70) than with NK-restricted markers (rho 0.54) (Table S5).

Substituting NK-restricted markers produced an apparently improved score (eight genes: HR 0.78, 95% CI 0.67–0.90, *p* = 0.0008, c = 0.565). Six of the eight are killer immunoglobulin-like receptors, four of which have median expression of zero across the cohort (Supplementary Table S6). Three observations complicate the interpretation of that signature (Table S4).

Firstly, the KIR component carries the association. Modeled jointly with NCR1 and KLRF1, the KIR block retained significance (HR 0.81, *p* = 0.038) while NCR1 and KLRF1 did not (HR 0.92, *p* = 0.35); the converse did not occur. The two blocks correlate only moderately (rho 0.62). Adding KIR improved model fit over NCR1 and KLRF1 alone (likelihood ratio χ^2^ = 4.75, 1 df, *p* = 0.029) while leaving discrimination unchanged (c 0.565 versus 0.563).

Discarding quantitative information improved the association. A simple count of how many of the six KIR genes were detected gave HR 0.86 per gene (95% CI 0.78–0.95, *p* = 0.0035), a stronger result than measured abundance (HR 0.82, *p* = 0.028). Sequencing depth does not explain this: the depth coefficient was essentially unity (HR 0.99, *p* = 0.97), though in a joint model containing depth and NK-restricted markers, both biological terms became marginal (KIR count *p* = 0.071; NCR1 and KLRF1 *p* = 0.084). The effect also plateaued: relative to samples with none or one KIR gene detected, hazard ratios were 0.73 for two, 0.54 for three, and 0.50 for four to six, with no meaningful increment beyond three.

The two KIR genes with substantive expression were sufficient on their own. KIR2DL4 and KIR3DL2 together gave HR 0.78 (95% CI 0.67–0.90, *p* = 0.0009, c = 0.557), equivalent to the full eight-gene signature and outperforming NCR1 and KLRF1.

#### Interaction Between NK Abundance and Tumor PD-L1 Expression

There was no evidence of interaction between NK abundance and tumor-cell PD-L1 (interaction HR 1.01, 95% CI 0.85–1.20, *p* = 0.91; NK main effect HR 0.81, *p* = 0.15; PD-L1 main effect HR 1.09, *p* = 0.40).

### 2.6. Clinical Covariates and Comparative Discrimination

After adjusting for available prognostic factors among 316 patients with complete clinical data (219 events), the NK association persisted (HR 0.80, 95% CI 0.66–0.97, *p* = 0.025), while the macrophage association attenuated (HR 1.17, *p* = 0.081). Both were far weaker than the clinical covariates: ECOG performance status, HR 1.73 (95% CI 1.35–2.21, *p* < 0.0001), and liver metastases, HR 1.61 (95% CI 1.21–2.14, *p* = 0.0011). This model showed the highest discrimination observed (c = 0.636). The adverse macrophage association attenuated after adjustment for performance status and liver metastases and is therefore less robust than the protective natural killer cell association, which persisted. We note, however, that liver metastasis may not be a simple confounder in this relationship: macrophage-mediated elimination of activated T cells has been proposed as the mechanism by which liver metastases restrain immunotherapy efficacy. If liver metastasis lies on the causal path between myeloid infiltration and adverse outcome, adjustment for it constitutes partial overadjustment, and the adjusted estimate may be conservative. We do not resolve this here, and the asymmetry between the two compartments should be weighed when interpreting the opposing-signals result.

Within the 213 patients with complete data for all variables, bootstrap confidence intervals for the concordance index were 0.518 (0.485–0.560) for the composite score, 0.597 (0.547–0.642) for tumor mutational burden, 0.570 (0.525–0.618) for the NK and macrophage model, and 0.625 (0.578–0.666) for performance status with liver metastases. The composite score was clearly inferior to the clinical model, whose intervals did not overlap; intervals for tumor mutational burden, the cell-type signatures, and the clinical covariates overlapped substantially, and we do not claim a ranking among them.

We could evaluate response data for 298 patients. Objective response rates were 22.4%, 16.7%, 25.7% and 26.0% at scores of 0, 1, 2 and 3, respectively, with no association between the composite and response after adjustment for trial cohort (odds ratio 1.11 per point, 95% CI 0.88–1.40, *p* = 0.38). The null therefore holds on a more proximal readout of drug effect than overall survival, which in platinum-refractory disease is confounded by subsequent therapy.

### 2.7. Multiplicity

After Benjamini-Hochberg correction across the ten exploratory models, the principal findings survived: the eight-gene signature (adjusted *p* = 0.008), NCR1 and KLRF1 (0.017), the KIR detection count (0.017), the initial NK signature (0.018), the suppression reversal (0.048) and the incremental contribution of KIR (0.048). Immune phenotype fell to marginal significance (0.053). The null results were unaffected.

### 2.8. Validation Using Established Deconvolution

Because we assembled the cell-type signatures by manual curation, we repeated the principal analyses using MCP-counter, an established deconvolution method. Estimates from this method correlated strongly with our signatures (NK, rho 0.91; macrophage versus monocytic lineage, rho 0.89) and reproduced the findings: NK cells were protective when modeled alone (HR 0.81, 95% CI 0.71–0.93, *p* = 0.0035) while monocytic lineage was uninformative (HR 0.99, *p* = 0.88); modeled jointly, both strengthened in opposite directions (NK HR 0.68, 95% CI 0.56–0.83, *p* = 0.0001; monocytic HR 1.27, 95% CI 1.06–1.52, *p* = 0.0079; concordance index 0.570).

Because MCP-counter resolves NK cells separately from CD8-positive T cells, we could directly assess the specificity of the NK association. In a model containing NK cells, CD8 T cells, and monocytic lineage, the NK association was unchanged (HR 0.66, 95% CI 0.54–0.82, *p* = 0.0001) while CD8 T cells contributed nothing (HR 1.06, *p* = 0.51) and monocytic lineage remained adverse (HR 1.27, *p* = 0.0096). The two lymphocyte estimates correlated only moderately (rho 0.58). The protective association is therefore attributable to NK cells rather than to cytotoxic lymphocyte infiltration in general.

### 2.9. Sensitivity Analyses

Repeating the joint model after quantile normalization gave concordant estimates (NK HR 0.72, 95% CI 0.61–0.85, *p* = 0.0001; macrophage HR 1.27, 95% CI 1.08–1.49, *p* = 0.003; concordance index 0.576), as did gene-wise standardization before signature averaging.

FCGR3A and FCGR3B correlated at rho 0.37, consistent with, but not demonstrative of, adequate separation of the two paralogues. Analyses restricted to the platinum-pretreated cohort (n = 272, 191 events) reproduced the principal findings. The composite score remained unassociated with overall survival (HR 1.02, 95% CI 0.91–1.15, *p* = 0.72, c-index 0.509). In the joint model, the NK and macrophage signatures again separated in opposite directions, with slightly larger effect estimates than in the pooled cohort (NK HR 0.68, 95% CI 0.56–0.81, *p* < 0.0001; macrophage HR 1.31, 95% CI 1.10–1.56, *p* = 0.0029; c-index 0.579). Because the transcriptomic release pools two clinically distinct populations, we repeated the principal models in the platinum-pretreated cohort alone as a sensitivity analysis.

## 3. Discussion

Multiple studies have sought biomarkers of immune checkpoint blockade benefit in advanced urothelial carcinoma, but none has been consistently adopted in routine practice. With this in mind, we evaluated the construct validity of a proposed CD16/IL-15/IL-15RA ADCC-competency score in patients treated with atezolizumab in the IMvigor210 phase II clinical trial, where ADCC cannot occur. The score was not associated with survival, but the reason differs from the one anticipated, and that difference carries more information than the null itself.

First, we found that the composite sums a protective NK compartment with an adverse macrophage compartment, and that the two mask one another. Analyzed separately, the NK signature gave a hazard ratio of 0.83 and the macrophage signature an apparently neutral 1.04; modeled together, they separated to 0.71 and 1.29. Macrophage infiltration in this cohort is not uninformative. It is adverse, and appears uninformative only when NK content is omitted. The possibility that opposing survival effects within an aggregated immune signature may cancel one another has been raised previously as a theoretical limitation of treating the immune infiltrate as a single quantity [21]; the present analysis provides direct empirical support. This pattern also has a precedent in the bladder cancer literature: a meta-analysis of tumor-associated macrophages found that CD68-defined macrophages showed no significant association with overall survival, whereas CD163-defined macrophages were significantly associated with worse recurrence-free survival [22]. A macrophage measure that appears uninformative in aggregate can therefore carry adverse prognostic weight once resolved, whether by marker choice or, as here, by adjustment for the compartment that masks it. Evidence also supports an adverse contribution of macrophages in this disease: liver metastases restrain immunotherapy efficacy through macrophage-mediated elimination of activated T cells [23].

The consequence for score construction is that any measure summing these compartments will read close to unity regardless of how much biology is present, because the components subtract. Increasing the score can mean more NK cells, more macrophages, or both, and these have opposite consequences for survival. The mechanism is unsurprising once stated: macrophages and neutrophils express FCGR3A, as do NK cells, and tumor and stromal cells express IL15RA, so a score built from these genes indexes a mixture of compartments, only one of which is the intended effector population. The consequence for tissue assays is immediate. Single-marker CD16 immunohistochemistry, which was the design of our original protocol, labels macrophages and neutrophils alongside NK cells and cannot separate them by morphology in routine practice; CD16 immunohistochemistry is subject to the same interpretive limitation as PD-L1, in that its result depends on what the antibody labels. Accordingly, we have proposed dual staining for CD16 with NKp46, scoring only double-positive cells. We emphasize that this protocol is proposed rather than validated: NKp46 is an established marker for NK cell identification [18], but we have not tested the combined stain on tissue, and CD16 shedding upon NK activation may complicate interpretation. This asymmetry is reflected in our own marker set. Three of the four genes comprising our macrophage signature (CD163, CSF1R, MRC1) are associated with alternative (M2) polarization rather than with macrophage abundance per se, so the adverse association we report is more accurately described as a signal of myeloid polarization than of myeloid content. We infer cell-type attribution throughout this work from marker expression in bulk tissue, and this is a statistically supported interpretation rather than a direct demonstration of cellular identity or localization.

Second, we characterized each component’s contribution by removing them in turn. Removing CD16 reduced the correlation between the composite and the macrophage signature from 0.80 to 0.69. It increased its correlation with NK-restricted markers from 0.50 to 0.55, whereas removing IL15 or IL15RA did not reduce the macrophage correlation (0.82 and 0.81, respectively). CD16 therefore contributes more myeloid signal to the composite than either interleukin-15 component. This corrects an interpretation we initially favored based on correlation magnitude alone: the high correlation between the IL-15 axis and the composite (rho 0.88) is largely arithmetic, since IL15 and IL15RA constitute two of three components. The revised interpretation better reflects the biology, as macrophages and neutrophils express FCGR3A in addition to NK cells, and the proposed immunohistochemical assay was based on this marker. The interleukin-15 components nonetheless carried no prognostic information individually or in combination (HR 0.99, *p* = 0.86), and IL-15 trans-presentation is a membrane protein–protein interaction that transcript abundance cannot capture in principle [19]. We therefore do not support inclusion of the IL-15 component in future versions of this score. No single-component removal recovered prognostic information (hazard ratios 0.95 to 0.98; all *p* > 0.45), indicating that the null association does not arise from one uninformative marker.

Third, we evaluated NK abundance in the absence of any ADCC mechanism. NK-restricted marker expression was associated with better survival in a cohort where ADCC cannot occur (HR 0.77, *p* = 0.001), and this association persisted after adjustment for performance status and liver metastases (HR 0.80, *p* = 0.025). This effect cannot be ADCC-mediated and reflects the established prognostic value of cytotoxic lymphocyte infiltration. The interaction analysis strengthens this reading: if NK abundance were exerting its effect through antibody-mediated killing, that effect should depend on the availability of target antigen for opsonization, yet we found no interaction between NK abundance and tumor-cell PD-L1 (HR 1.01, *p* = 0.91). This has important implications for future work and parallels a difficulty already documented for PD-L1 itself. In the control arm of JAVELIN Bladder 100, PD-L1-positive patients survived longer than the overall population, suggesting PD-L1 has a prognostic component in that setting and is not purely predictive. NK abundance behaves similarly here. If a composite ADCC score later shows benefit in an avelumab-treated cohort, that finding will be uninterpretable unless the score outperforms NK abundance alone and is evaluated in a design that can separate prognostic from predictive effects. Because JAVELIN Bladder 100 is randomized, the appropriate analysis is a treatment-by-biomarker interaction across both arms rather than an association within the treated arm.

Fourth, the NK-restricted signature we constructed proved dominated by killer immunoglobulin-like receptor (KIR) genes, and its behavior is not straightforward. Two features suggest it may index something other than cell abundance. A count of how many KIR genes were detected at all outperformed their measured abundance, and the effect plateaued after three genes. A marker of infiltration should not improve when quantitative information is discarded, nor cease responding once a threshold is passed. KIR genes vary between individuals in germline gene content, not only in expression level [24], and both features are consistent with what such variation would produce in tumor RNA sequencing. Against this, the two KIR genes with substantive expression were sufficient on their own, performing as well as the full signature, which is what a genuine NK-associated transcript signal would produce. We cannot resolve this without germline genotyping and do not claim to have demonstrated a germline effect. What we can say is that a signature built from a polymorphic gene family behaved in ways abundance alone does not explain, and that we would not have detected this had we not asked whether presence outperformed abundance. When a signature draws on polymorphic loci such as KIR, HLA, immunoglobulin, or Fcγ receptor genes, analyze presence and abundance separately, and inspect dose-response for plateaus, before interpreting the signature as a measure of infiltration.

Finally, when characterizing the score’s behavior under adjustment, we observed that adding immune phenotype and PD-L1 reversed its coefficient and produced nominal significance. Because the same reversal appeared in the identical patients without adjustment, and because the score correlates strongly with both adjusters, this is best understood as suppression under collinearity rather than a biological effect. Reporting such a coefficient as a finding would be an instance of the Table 2 fallacy [25]. We note this explicitly because immune biomarker studies frequently adjust for correlated immune covariates and then interpret the resulting coefficients directly. ADAM17 also sheds CD16 protein upon NK activation [26], so transcript abundance may diverge from protein detection in activated cells, a consideration relevant to translating these findings to immunohistochemistry.

The assay dependence that motivated this work merits emphasis, because it is not a minor technical caveat. In direct comparison, the Dako 73-10 protocol used in the avelumab development program stains a greater proportion of tumor cells than 22C3, 28-8, or SP263, whereas the Ventana SP142 assay used in IMvigor210 stains fewer [11]. As a result, the same tumor may be assigned opposite PD-L1 categories depending on the reagent and scoring algorithm used, and patient populations defined as PD-L1-positive across different trials are not equivalent. This is why we judged a target-side biomarker insufficient at the outset of this work, and why single-marker CD16 immunohistochemistry, which labels macrophages and neutrophils alongside NK cells, does not escape the same class of problem.

### 3.1. Discrimination and Clinical Utility

One measurement constrains all of the above. Across the composite score, the individual genes, all cell-type signatures, the KIR analyses, and every combination examined, the concordance index never exceeded 0.588. No model approached the discrimination required for patient selection. The composite score performed no better than chance and was clearly inferior to a model using performance status and liver metastases, with nonoverlapping confidence intervals. At this sample size, we could not resolve differences among tumor mutational burden, the cell-type signatures, and the clinical covariates, and we do not claim a ranking among them.

These findings should be considered against clinical prognostic models already developed for this setting, several of which integrate performance status, liver metastases, and routine laboratory variables [27,28,29,30]. In our analysis, the clinical model achieved a concordance index of 0.625 (95% CI 0.578–0.666), exceeding that of the composite score but overlapping with those of tumor mutational burden and the cell-type signatures. Several of the associations we report are statistically robust and survive multiplicity correction. Yet none approaches the discrimination required for patient selection, and the distinction between statistical significance and discriminatory capacity is well recognized [31]. Whether effector-side molecular biomarkers can add to such clinical models, and at what cost and complexity, requires benchmarking studies that directly evaluate single and combined predictors. A comparable retrospective validation in this cohort has shown that a molecular score can retain independent prognostic significance alongside performance status and liver metastases [32], indicating that the question is tractable rather than settled.

### 3.2. Limitations

Our study has four main limitations. First, we used a cohort from a single-arm phase II clinical trial. Because there was no comparator arm, we could not test for interaction to determine whether any of the signatures evaluated are predictive rather than prognostic. Validation in a cohort from a randomized phase III clinical trial could clarify this point.

The second limitation concerns the assumption underlying the negative-control design. Atezolizumab and avelumab differ in more than Fc effector function: they differ in antigen-binding characteristics, in the clinical settings in which they have been evaluated, and in the trial populations from which the available data derive. Attribution of the observed null association specifically to the absence of ADCC therefore assumes that Fc competence is the operative difference between the two agents, and this assumption cannot be verified within a single-arm cohort treated with only one of them. Any of these unmeasured differences could contribute to the null.

The third is a mismatch in clinical setting. JAVELIN Bladder 100 evaluated maintenance therapy in patients who had responded to platinum, whereas this dataset comprises platinum-refractory and cisplatin-ineligible first-line patients. Chemotherapy alters the tumor immune microenvironment, so a signature’s behavior here may not transfer to the maintenance setting. Relatedly, the transcriptomic release pools two biologically distinct populations: 76 platinum-naive patients and 272 whose tumors had been exposed to platinum. The immune signatures analyzed here are therefore not measured under equivalent conditions in the two groups. Stratification by cohort allows each group its own baseline hazard. However, this does not address differences in underlying biology, and the cohorts also differed materially in performance status and primary tumor site (Table 1). Analyses restricted to the 272 platinum-pretreated patients gave concordant estimates: the composite score remained unassociated with survival (HR 1.02, 95% CI 0.91–1.15, *p* = 0.72), while in the joint model the NK and macrophage signatures separated in opposite directions with slightly larger effect sizes than in the pooled cohort (NK HR 0.68, 95% CI 0.56–0.81, *p* < 0.0001; macrophage HR 1.31, 95% CI 1.10–1.56, *p* = 0.0029; concordance index 0.579). The principal findings therefore do not depend on pooling the two cohorts, although residual confounding by treatment exposure cannot be excluded.

The fourth limitation is that all analyses of the NK-restricted signature are post-hoc. They were prompted by inspection of the detection table rather than pre-specified; the categorical model used boundaries chosen after the continuous result was known; and germline gene content was inferred from tumor RNA detection rather than genotyped. These analyses should therefore be regarded as hypothesis-generating, and independent replication with germline data is required before these observations can be considered established.

The principal limitation of this work is its reliance on bulk transcriptomic data as a surrogate for a proposed immunohistochemical assay. Bulk expression cannot establish cell-type localization, confirm protein-level expression, or capture the spatial relationship between effector and target cells required for antibody-dependent cellular cytotoxicity. Attribution of signal to particular cell types therefore rests on inference from marker expression rather than demonstrated cellular origin, and concordance between transcript and protein measurements has not been established in matched material.

FCGR3A and FCGR3B are highly homologous, and the neutrophil-restricted FCGR3B could contribute to apparent FCGR3A signal. We derived quantification from the published processing pipeline for this dataset, and alignment-level data permitting assessment of multimapping between these genes were not available to us.

Several further considerations apply. PD-L1 in this cohort was scored with the Ventana SP142 assay, a third assay distinct from both the SP263 of JAVELIN Bladder 100 and the Dako 73-10 of the avelumab development program, so our PD-L1 results are assay-specific. Hemoglobin was unavailable, so clinical adjustment was incomplete, and 32 patients lacked performance status or metastatic site data. NCR1 and KLRF1 are also expressed by group 1 innate lymphoid cells, so the two-gene signature is more accurately described as NK/ILC1-restricted. Overall survival in platinum-refractory disease is confounded by subsequent therapy, which was not recorded. Finally, the premise motivating this work, that ADCC contributes materially to avelumab benefit, remains unproven; the FcγR allele association in JAVELIN Bladder 100 was exploratory, and the comparable clinical activity of effector-silenced anti-PD-L1 agents in urothelial carcinoma suggests any ADCC contribution may be modest [15].

## 4. Materials and Methods

### 4.1. Study Design and Patient Population

We analyzed the IMvigor210 dataset, which contains RNA sequencing and clinical outcome data for 348 patients with locally advanced or metastatic urothelial carcinoma treated with atezolizumab monotherapy. We performed all analyses in R version 4.6.1 on Windows, using survival 3.8-9, edgeR 4.10.1, Biobase 2.72.0, limma 3.68.4, car 3.1-5, and MCPcounter (GitHub, ebecht/MCPcounter; no versioned release).

A practical note on access is warranted because it affects reproducibility. The package stores its data as a CountDataSet, a class defined by the first version of the DESeq package. DESeq version 1 has been retired from Bioconductor and cannot be installed under current versions of R, so the package’s accessor functions fail. We therefore extracted the count matrix, phenotype table, and feature annotation directly from the object’s storage slots using base R attribute access, which does not require loading the class definition. We confirmed the extracted matrices contained 31,286 genes and 348 samples before any analysis. Investigators attempting to reproduce this work on current software will encounter the same obstacle.

The transcriptomic release combines patients from both IMvigor210 cohorts. We identified cohort membership from recorded prior platinum exposure, yielding 76 platinum-naive patients (cohort 1, cisplatin-ineligible, first line) and 272 platinum-pretreated patients (cohort 2, platinum-refractory). At the time of analysis, 232 deaths had occurred (66.7%).

Atezolizumab carries an Fc region engineered to eliminate effector function. Any biomarker specific to antibody-dependent cellular cytotoxicity (ADCC) should therefore be inert in this cohort. We selected the cohort for this property, not to test the primary hypothesis.

This is a secondary analysis of previously published, publicly available, and fully de-identified data. No new patient material was collected, and no individual patient can be identified from the data used, so institutional review board approval and informed consent were not required for the present analysis. The primary IMvigor210 trial was conducted in accordance with the Declaration of Helsinki and Good Clinical Practice guidelines, was approved by the institutional review board or independent ethics committee at each participating site, and all participants gave written informed consent. We report the study in accordance with the REMARK recommendations for tumor marker prognostic studies [33]; a completed checklist appears as Supplementary Material.

We converted raw counts to counts per million using edgeR::cpm() and log2-transformed them after adding a pseudocount of one. We used log2 counts per million because signatures were computed by averaging marker expression within each sample, and this scale is standard for gene-set scoring of bulk RNA-sequencing data; we assessed robustness to alternative normalization separately (Section 2.9). We labeled rows with gene symbols from the package’s feature annotation. We performed all analyses in R version 4.6.1 on Windows, using survival 3.8-9, edgeR 4.10.1, and Biobase 2.72.0. The analysis session used Turkish locale collation, which orders dotted and dotless characters differently from English.

### 4.2. The Composite Score

The immunohistochemical protocol was under development at the time of this analysis and had not been finalized; specific antibody clones and scoring thresholds had not been fixed. The transcriptomic composite was therefore constructed to reflect the intended structure of that assay—three markers, each dichotomized and summed—rather than to replicate a defined scoring system. We computed medians across the pooled cohort rather than within trial cohorts, so the score corresponds to a single fixed threshold as a tissue assay would apply; values exactly equal to the median were assigned to the low category. Because dichotomization discards information and cohort-derived medians do not transfer across cohorts, we also analyzed a continuous version of the composite, defined as the mean z-score of the three genes [34].

### 4.3. Cell-Type Signatures

We computed signatures as the mean log2 counts per million across defined marker sets. The initial natural killer (NK) cell signature comprised NCR1, KLRD1, NKG7, GNLY, PRF1, and KLRF1. We selected these genes through literature-based curation of markers conventionally used to identify NK cells and their cytotoxic effector program, rather than adopting a single published signature: NCR1 and KLRF1 encode NK-restricted activating receptors, KLRD1 encodes CD94, and NKG7, GNLY, and PRF1 encode components of the cytotoxic granule machinery [35]. We acknowledge that this selection is subjective. Several published NK gene signatures exist, and different marker sets would be expected to yield somewhat different estimates; we therefore examine the sensitivity of our findings to signature composition by analyzing component subsets (Supplementary Tables S4 and S6), rather than assuming a single signature. Because three of these genes—NKG7, GNLY, and PRF1—are also expressed by CD8-positive T cells, we describe that set as a cytotoxic lymphocyte signature, and we analyzed a restricted two-gene signature of NCR1 and KLRF1 alongside it wherever the distinction mattered—the macrophage signature comprised CD68, CD163, CSF1R, and MRC1. We defined the interleukin-15 (IL-15) axis as the mean of IL15 and IL15RA. We indexed total leucocyte content by PTPRC (CD45).

### 4.4. NK-Restricted Markers

We constructed an NK-restricted signature to reduce contamination from other cell types. The final set comprised eight genes: KIR2DL1, KIR2DL3, KIR2DL4, KIR3DL1, KIR3DL2, KIR3DS1, NCR1 and KLRF1. Canonical killer immunoglobulin-like receptor genes were selected by manual curation. This excluded KIRREL, KIRREL2 and KIRREL3, which are nephrin-family adhesion molecules unrelated to killer immunoglobulin-like receptors despite their symbols, and the divergent KIR3DX1. A gene was considered detected in a sample when at least one read mapped to it (Supplementary Table S6); genes were selected for the signature by manual curation of canonical killer immunoglobulin-like receptors rather than by applying a detection threshold. We analyzed KIR components separately: the two KIR genes with substantive expression (KIR2DL4, median log2 CPM 0.33; KIR3DL2, 0.13), the four with zero median expression (KIR2DL1, KIR2DL3, KIR3DL1, KIR3DS1), and the two non-KIR markers (NCR1, 0.58; KLRF1, 0.57). To separate transcript abundance from receptor repertoire composition, we also computed a detection count: the number of the six KIR genes with at least one mapped read in a given sample, an integer from 0 to 6. Every analysis involving the NK-restricted set, its components, and the detection count is post-hoc. These analyses were prompted by inspection of the detection table rather than pre-specified, and the resulting estimates are optimistically biased because they were developed in the same data.

### 4.5. Statistical Analysis

Overall survival was defined as the interval from treatment initiation to death from any cause and analyzed by Cox proportional hazards regression using coxph(). Because the dataset combines two clinically distinct populations, we stratified all models by trial cohort using strata(). Stratification allows each cohort its own baseline hazard while estimating a single common covariate effect.

We also adjusted for the prognostic factors available in the public release: baseline ECOG performance status and liver metastases. Hemoglobin concentration, the third component of the standard risk model in this disease, is not recorded in this dataset and could not be included.

We standardized continuous predictors with scale(), so hazard ratios reflect the effect of a one-standard-deviation increase and are directly comparable across signatures. For the four ordered score groups, we report the trend test—a Cox model with the score entered as a continuous variable—rather than the four-group log-rank test, which spends three degrees of freedom against an ordered alternative and has correspondingly lower power.

Where we asked whether one signature added information to another, we compared nested models with a likelihood ratio test using anova() and also reported the difference in Harrell’s concordance index. We assessed the proportional hazards assumption with scaled Schoenfeld residuals via cox.zph(). We used Spearman rank correlations between signatures. We analyzed objective response with logistic regression.

We included library sequencing depth, computed as the column totals of the raw count matrix, as a covariate in analyses of the KIR detection count.

We designated the trend test for the composite score and its continuous hazard ratio as the primary analyses. All other models are exploratory. We report nominal *p*-values throughout and also provide Benjamini-Hochberg adjusted values for the exploratory family. The exploratory family comprised the ten survival models testing an association between a candidate marker or signature and overall survival. We did not include sensitivity and validation analyses that re-estimate an existing association under alternative preprocessing or deconvolution, because they test the robustness of a single hypothesis rather than additional hypotheses. We designated analyses as pre-specified or exploratory as follows: we defined the composite score, its three components, and the cell-type signatures before any survival analysis, whereas we defined the NK-restricted marker set, the KIR component analyses, and the detection-count measure after inspecting gene detection rates, and we treated them as exploratory throughout. We handled missing data with complete-case analysis and reported the number of patients and events for each model.

To assess whether the cell-type interpretation depended on our marker choice, we repeated the principal analyses using MCP-counter, an established deconvolution method. To assess robustness to preprocessing, we also repeated them using quantile normalization between samples and, separately, gene-wise standardization before signature averaging. We computed variance inflation factors for the joint model. We compared concordance indices within the subset of patients with complete data for all relevant variables, with confidence intervals obtained by bootstrap resampling with 1000 replicates.

## 5. Conclusions

A CD16/IL-15/IL-15RA composite reads null under effector-silenced PD-L1 blockade, not because it is specific for ADCC but because its components carry opposing associations that mask one another within a summed score: a protective NK signal and an adverse macrophage signal. Component-wise analysis identified CD16, rather than the interleukin-15 axis, as the main source of myeloid weighting, and an established deconvolution method reproduced the opposing associations. Replace single-marker CD16 assessment with NK-restricted markers or dual staining, and reconsider the interleukin-15 components, which carry no prognostic information. Because NK abundance is prognostic where ADCC cannot occur, and showed no evidence of interaction with tumor PD-L1 expression, future ADCC-competency scores must demonstrate incremental value over NK abundance alone in a randomized design before any predictive claim is made. Across every model tested, discrimination remained near chance and below what performance status and metastatic site alone achieve, setting a demanding benchmark for effector-side biomarkers in this disease.

## Figures and Tables

**Figure 1:**
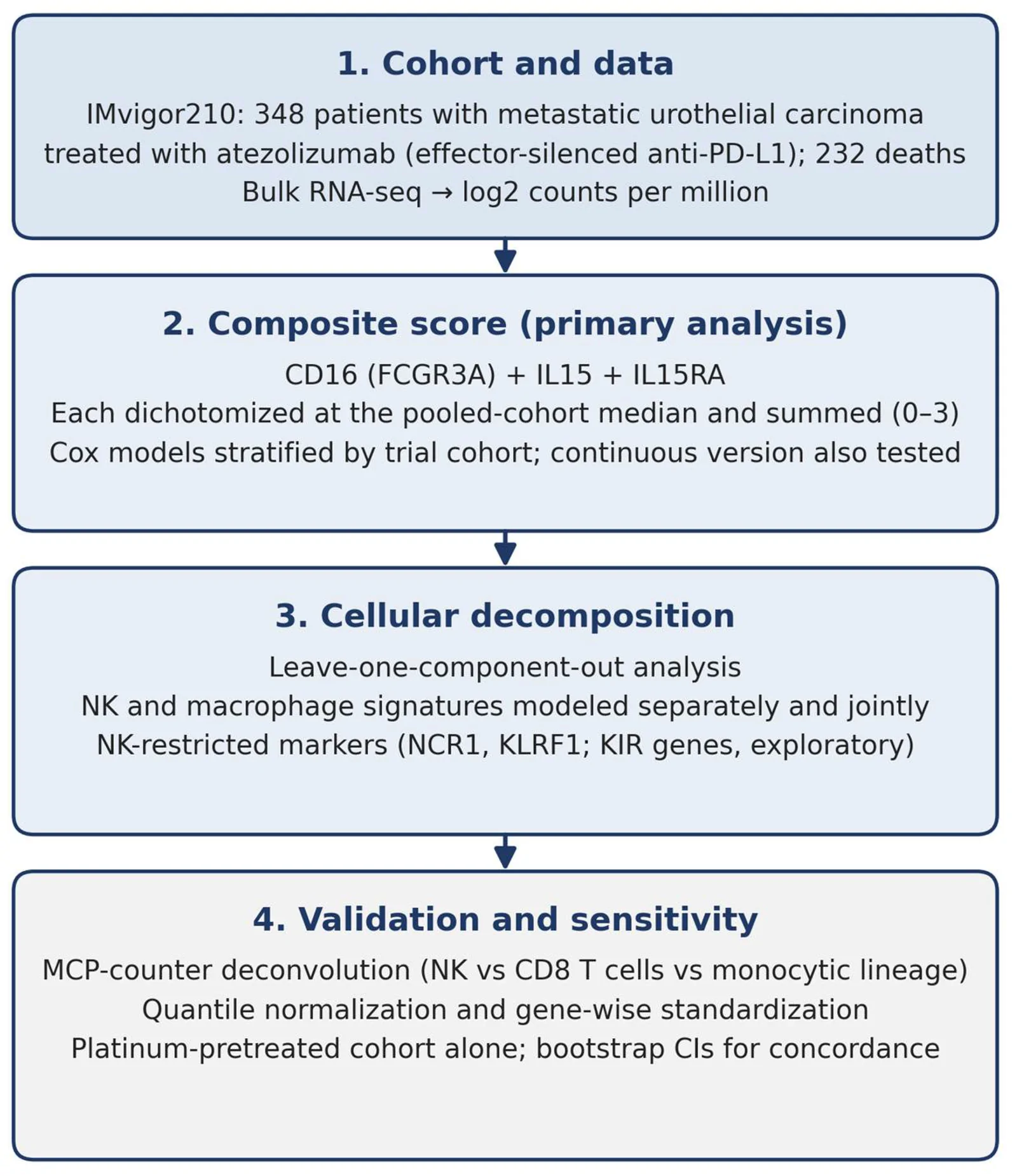
Analytical workflow. We constructed the CD16/IL-15/IL-15RA composite score from bulk transcriptomic data in the IMvigor210 cohort and evaluated it as the primary analysis, then decomposed it into cellular compartments and validated it with an established deconvolution method and alternative normalization. CI: confidence interval; KIR: killer immunoglobulin-like receptor; NK: natural killer.

**Figure 2:**
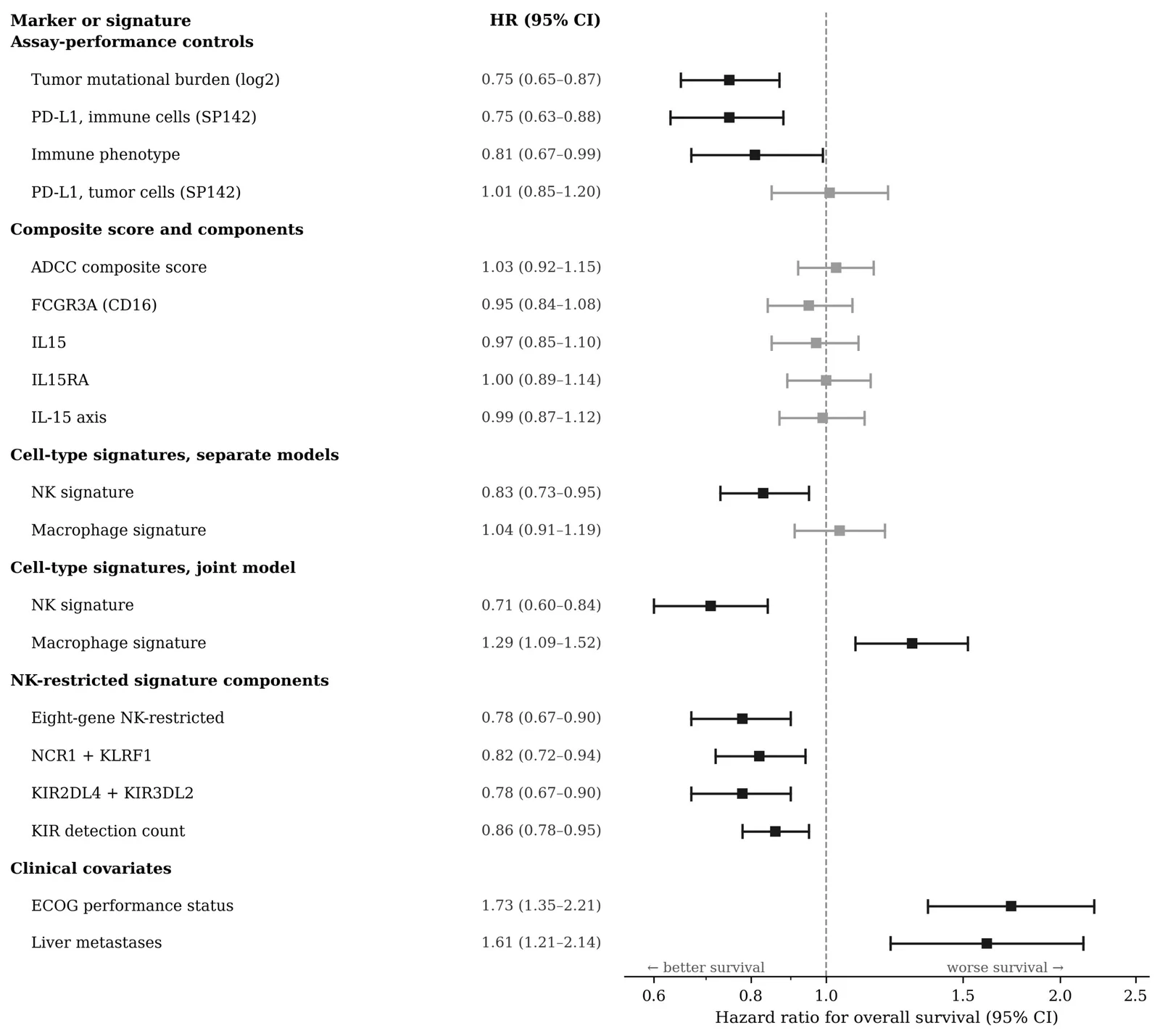
Forest plot of hazard ratios for overall survival with 95% confidence intervals across all markers and signatures evaluated. All estimates come from Cox proportional hazards models stratified by trial cohort; continuous predictors are standardized, so hazard ratios reflect the effect of a one-standard-deviation increase. Black markers indicate confidence intervals excluding unity; grey markers indicate those including unity. Note that the NK and macrophage signatures separate in opposite directions when modeled jointly, and that the clinical covariates show larger effects than any transcriptomic signature. ADCC: antibody-dependent cellular cytotoxicity; CI: confidence interval; ECOG: Eastern Cooperative Oncology Group; HR: hazard ratio; NK: natural killer; PD-L1: programmed death-ligand 1.

**Table 1:** Baseline characteristics of the IMvigor210 transcriptomic cohort.

Variable	Cohort 1 (*n* = 76)	Cohort 2 (*n* = 272)	Total (*n* = 348)
**Sex, *n* (%)**			
Female	14 (18.4)	62 (22.8)	76 (21.8)
Male	62 (81.6)	210 (77.2)	272 (78.2)
**ECOG performance status, *n* (%)**			
0	23 (30.3)	111 (40.8)	134 (38.5)
1	36 (47.4)	160 (58.8)	196 (56.3)
2	17 (22.4)	1 (0.4)	18 (5.2)
**Metastatic disease status, *n* (%)** ^a^			
Liver	18 (26.9)	80 (32.1)	98 (31.0)
Lymph node only	16 (23.9)	44 (17.7)	60 (19.0)
Other visceral	33 (49.3)	125 (50.2)	158 (50.0)
**Tissue site, *n* (%)** ^b^			
Bladder	38 (51.4)	157 (59.0)	195 (57.4)
Renal pelvis	22 (29.7)	45 (16.9)	67 (19.7)
Ureter	8 (10.8)	18 (6.8)	26 (7.6)
Lymph node	4 (5.4)	22 (8.3)	26 (7.6)
Other	2 (2.7)	24 (9.0)	26 (7.6)
**PD-L1, immune cells (SP142), *n* (%)** ^c^			
IC0	19 (25.0)	78 (28.8)	97 (27.9)
IC1	36 (47.4)	96 (35.4)	132 (38.0)
IC2+	21 (27.6)	97 (35.8)	118 (34.0)
**PD-L1, tumor cells (SP142), *n* (%)** ^c^			
TC0	59 (77.6)	216 (79.7)	275 (79.3)
TC1	8 (10.5)	14 (5.2)	22 (6.3)
TC2+	9 (11.8)	41 (15.1)	50 (14.4)
**TCGA subtype, *n* (%)**			
I	20 (26.3)	98 (36.0)	118 (33.9)
II	23 (30.3)	72 (26.5)	95 (27.3)
III	20 (26.3)	49 (18.0)	69 (19.8)
IV	13 (17.1)	53 (19.5)	66 (19.0)
**Data availability, *n* (%)**			
Tumor mutational burden	70 (92.1)	202 (74.3)	272 (78.2)
Immune phenotype	61 (80.3)	223 (82.0)	284 (81.6)
PD-L1	76 (100)	271 (99.6)	347 (99.7)
Metastatic disease status	67 (88.2)	249 (91.5)	316 (90.8)
**Outcome**			
Deaths, *n* (%)	41 (53.9)	191 (70.2)	232 (66.7)
Median overall survival, months (95% CI)	14.13 (7.72–NR)	8.02 (6.74–10.3)	8.79 (7.72–10.3)

Cohort 1: cisplatin-ineligible, treatment-naive. Cohort 2: platinum-pretreated. Percentages are calculated among patients with non-missing data for each variable. Age at diagnosis, prior lines of therapy, and hemoglobin concentration are not recorded in the public data release. ^a^ Missing for 32 patients (9 in cohort 1, 23 in cohort 2). ^b^ Missing for 8 patients; “other” combines lung, liver, and unspecified sites. ^c^ Missing for 1 patient. CI: confidence interval; ECOG: Eastern Cooperative Oncology Group; NR: not reached; PD-L1: programmed death-ligand 1; TCGA: The Cancer Genome Atlas.

**Table 2:** Overall survival by composite score.

ADCC Score	*n*	Deaths	Median OS, Months (95% CI)
0	96	60	9.23 (7.39–15.3)
1	76	53	8.08 (6.51–13.3)
2	82	55	7.85 (5.65–14.1)
3	94	64	9.76 (6.60–12.7)

Continuous score, stratified by trial cohort: HR 1.03 (95% CI 0.92–1.15), *p* = 0.64, c-index 0.510. Four-group log-rank: chi-square 0.5, 3 degrees of freedom, *p* = 0.9. CI: confidence interval; OS: overall survival.

**Table 3:** Composite score versus tumor immune phenotype.

ADCC Score	*n*	Desert	Excluded	Inflamed
0	79	39 (49.4)	35 (44.3)	5 (6.3)
1	59	20 (33.9)	32 (54.2)	7 (11.9)
2	68	11 (16.2)	40 (58.8)	17 (25.0)
3	78	6 (7.7)	27 (34.6)	45 (57.7)

Values are *n* (row %). Immune phenotype was available for 284 of 348 patients. Spearman correlation with the score: 0.50. ADCC: antibody-dependent cellular cytotoxicity.

**Table 4:** Cell-type decomposition of the composite score.

Model	Signature	HR (95% CI)	*p*	c-Index
Separate	NK (initial set)	0.83 (0.73–0.95)	0.0071	0.554
Separate	Macrophage	1.04 (0.91–1.19)	0.56	0.505
Separate	IL-15 axis	0.99 (0.87–1.12)	0.86	0.503
Joint	NK (initial set)	0.71 (0.60–0.84)	0.0001	0.575
Joint	Macrophage	1.29 (1.09–1.52)	0.0024	0.575
Joint	NK-restricted (NCR1, KLRF1)	0.77 (0.67–0.90)	0.001	0.572
Joint	Macrophage	1.15 (1.00–1.32)	0.056	0.572

*n* = 348 with 232 events throughout, stratified by trial cohort. Hazard ratios are per one standard deviation increase. Initial NK set: NCR1, KLRD1, NKG7, GNLY, PRF1, KLRF1. Macrophage set: CD68, CD163, CSF1R, MRC1. CI: confidence interval; HR: hazard ratio; IL-15: interleukin-15; NK: natural killer.

## Data Availability

All clinical and molecular tumor data from the IMvigor210 trial used for this retrospective study have been previously deposited in the European Genome-Phenome Archive under accession number EGAS00001002556 and are freely available through the IMvigor210CoreBiologies R package (http://research-pub.gene.com/IMvigor210CoreBiologies, accessed on 4 August 2026), version 1.0.1 [8]. The Supplementary Tables and Figures are deposited at 10.5281/zenodo.22690672, and the analysis code is available from the corresponding author upon reasonable request.

## Supplementary Materials

The following supporting information can be downloaded at: https://www.mdpi.com/article/10.3390/ijms27208943/s1.

## Abbreviations

The following abbreviations are used in this manuscript:

ADCC	Antibody-dependent cellular cytotoxicity
CI	Confidence interval
CPM	Counts per million
ECOG	Eastern Cooperative Oncology Group
HR	Hazard ratio
IC	Immune cell
IHC	Immunohistochemistry
IL-15	Interleukin-15
IL-15RA	Interleukin-15 receptor alpha
KIR	Killer immunoglobulin-like receptor
NK	Natural killer
OS	Overall survival
PD-L1	Programmed death-ligand 1
REMARK	Reporting Recommendations for Tumor Marker Prognostic Studies
TC	Tumor cell
TCGA	The Cancer Genome Atlas
