# Supplementary material for: Opposing Natural Killer and Macrophage Associations Are Masked Within a CD16/IL-15/IL-15RA Composite Score in Metastatic Urothelial Carcinoma
Source: Int J Mol Sci. 2026 Oct 9;27(20):8943. doi: 10.3390/ijms27208943 (PMC13704327; doi:10.3390/ijms27208943)
Supplement: Supplementary file 1 [file ijms-27-08943-s001.zip › ijms-4588171-supplementary.pdf]

Supplementary Materials

Opposing natural killer and macrophage associations are masked within a CD16/IL-15/IL-15RA composite score in metastatic urothelial carcinoma

Supplementary Table S1. Assay-performance controls analyzed through the identical pipeline.

**Marker**	**n**	**Events**	**HR (95% CI)**	**p**	**c-index**
log2 tumor mutational burden	272	173	0.75 (0.65-0.87)	0.0001	0.588
PD-L1, immune cells (SP142)	347	232	0.75 (0.63-0.88)	0.0004	0.565
Immune phenotype (desert to inflamed)	284	190	0.81 (0.67-0.99)	0.037	0.541
PD-L1, tumor cells (SP142)	347	232	1.01 (0.85-1.20)	0.92	0.506
ADCC-competency score	348	232	1.03 (0.92-1.15)	0.64	0.510

All models stratified by trial cohort. ADCC: antibody-dependent cellular cytotoxicity; CI: confidence interval; HR: hazard ratio; PD-L1: programmed death-ligand 1.

Supplementary Table S2. Individual component genes.

**Gene**	**HR (95% CI)**	**p**	**c-index**
FCGR3A (CD16)	0.95 (0.84-1.08)	0.46	0.501
IL15	0.97 (0.85-1.10)	0.61	0.512
IL15RA	1.00 (0.89-1.14)	0.95	0.496

n=348 with 232 events for all models, stratified by trial cohort. Hazard ratios are per one-standard-deviation increase. CI: confidence interval; HR: hazard ratio.

Supplementary Table S3. Coefficient reversal under adjustment for correlated immune covariates.

Model	n	HR for score (95% CI)	p
Score alone, full cohort	348	1.03 (0.92-1.15)	0.64
Score alone, complete cases	284	1.02 (0.90-1.16)	0.73
Score + immune phenotype + PD-L1 IC	284	1.18 (1.02-1.37)	0.028

In the adjusted model, PD-L1 on immune cells gave HR 0.73 (p=0.010) and immune phenotype HR 0.87 (p=0.27); c-index 0.570. The score correlates with immune phenotype (0.50) and with PD-L1 on immune cells (0.44). Adjusted coefficients are not interpreted causally. CI: confidence interval; HR: hazard ratio; IC: immune cell.

Supplementary Table S4. Cell-type decomposition of the composite score.

Model	Signature	HR (95% CI)	p	c-index
Separate	NK (initial set)	0.83 (0.73-0.95)	0.0071	0.554
Separate	Macrophage	1.04 (0.91-1.19)	0.56	0.505
Separate	IL-15 axis	0.99 (0.87-1.12)	0.86	0.503
Joint	NK (initial set)	0.71 (0.60-0.84)	0.0001	0.575
Joint	Macrophage	1.29 (1.09-1.52)	0.0024	0.575
Joint	NK-restricted (NCR1, KLRF1)	0.77 (0.67-0.90)	0.001	0.572
Joint	Macrophage	1.15 (1.00-1.32)	0.056	0.572

n=348 with 232 events throughout, stratified by trial cohort. Hazard ratios are per one standard deviation increase. Initial NK set: NCR1, KLRD1, NKG7, GNLY, PRF1, KLRF1. Macrophage set: CD68, CD163, CSF1R, MRC1. CI: confidence interval; HR: hazard ratio; IL-15: interleukin-15; NK: natural killer.

Supplementary Table S5. Spearman correlations between the composite score and cell-type signatures.

	Composite	NK initial	NCR1/KLRF1	Macrophage	IL-15 axis
Composite score	1.00	0.70	0.47	0.75	0.88
NK initial set	0.70	1.00	0.79	0.65	0.77
NCR1 + KLRF1	0.47	0.79	1.00	0.42	0.54
Macrophage	0.75	0.65	0.42	1.00	0.70
IL-15 axis	0.88	0.77	0.54	0.70	1.00
Immune phenotype	0.50	0.60	0.35	0.42	0.53

Values are Spearman rank correlation coefficients. The composite tracks the macrophage signature more closely than NK-restricted markers. Its high correlation with the IL-15 axis is largely arithmetic, because IL15 and IL15RA are two of its three components; leave-one-component-out analysis identifies CD16 as the main source of myeloid weighting (main text, Section 2.3). IL-15: interleukin-15; NK: natural killer.

Supplementary Table S6. Detection rates and expression levels of candidate natural killer cell markers in the IMvigor210 transcriptomic cohort (n=348).

Gene	Detection rate, %	Median log2 CPM	Included in signature	Reason for exclusion
KIRREL	100.0	4.872	No	Nephrin-family adhesion molecule, unrelated to KIR
NCR1	97.4	0.581	**Yes**	—
KLRF1	97.1	0.572	**Yes**	—
KIR2DL4	91.4	0.327	**Yes**	—
KIR3DL2	81.3	0.134	**Yes**	—
KIRREL3	80.2	0.113	No	Nephrin-family adhesion molecule, unrelated to KIR
KIR3DX1	67.8	0.077	No	Divergent KIR-like gene, not a canonical KIR
KIRREL2	63.8	0.042	No	Nephrin-family adhesion molecule, unrelated to KIR
KIR2DL3	53.2	0.029	**Yes**	—
KIR3DL1	52.9	0.028	**Yes**	—
KIR2DL1	33.3	0.000	**Yes**	—
KIR3DL3	14.9	0.000	No	Canonical KIR; not retained at curation
KIR3DS1	13.2	0.000	**Yes**	—
KIR2DS5	7.8	0.000	No	Canonical KIR; not retained at curation
KIR2DS1	5.2	0.000	No	Canonical KIR; not retained at curation
KIR2DS2	4.9	0.000	No	Canonical KIR; not retained at curation
KIR2DL2	4.9	0.000	No	Canonical KIR; not retained at curation
KIR2DS4	0.9	0.000	No	Canonical KIR; not retained at curation

Gene	Detection rate, %	Median log2 CPM	Included in signature	Reason for exclusion
KIRREL3-AS3	0.3	0.000	No	Antisense transcript, nephrin-family locus
KIRREL3-AS2	0.0	0.000	No	Antisense transcript, nephrin-family locus
KIR2DS3	0.0	0.000	No	Not detected in any sample
KIR2DL5B	0.0	0.000	No	Not detected in any sample
KIR2DL5A	0.0	0.000	No	Not detected in any sample

Genes are ordered by detection rate. Detection rate is the percentage of the 348 samples with at least one mapped read. Candidate genes comprised all symbols matching “KIR” in the feature annotation, together with NCR1 and KLRF1. The eight genes forming the NK-restricted signature were selected by manual curation of canonical killer immunoglobulin-like receptors; this excluded KIRREL, KIRREL2, KIRREL3 and their antisense transcripts, which are nephrin-family adhesion molecules unrelated to killer immunoglobulin-like receptors despite their symbols, and the divergent KIR3DX1.

No monotone detection threshold reproduces the selected set: KIR3DS1 was included at a detection rate of 13.2% while KIR3DL3 was excluded at 14.9%. Selection was therefore by curation rather than by application of a detection filter, and this is stated in the Methods.

Five canonical killer immunoglobulin-like receptor genes (KIR2DS5, KIR2DS1, KIR2DS2, KIR2DL2 and KIR2DS4) and KIR3DL3 were not retained during curation. These genes are canonical receptors and were not excluded on principled grounds evident from the data; the specific rationale applied at the time of curation was not recorded. All six were detected in fewer than 15% of samples. This is reported for transparency, and the sensitivity of the reported associations to signature composition is addressed in the component analyses reported in Section 2.5 of the main text.

Four of the six KIR genes in the signature (KIR2DL1, KIR2DL3, KIR3DL1, KIR3DS1) have a median log2 CPM of zero, indicating absent or near-absent expression in more than half of samples. This observation motivated the post-hoc component analyses reported in Section 2.5 of the main text.

CPM: counts per million; KIR: killer immunoglobulin-like receptor; NK: natural killer.

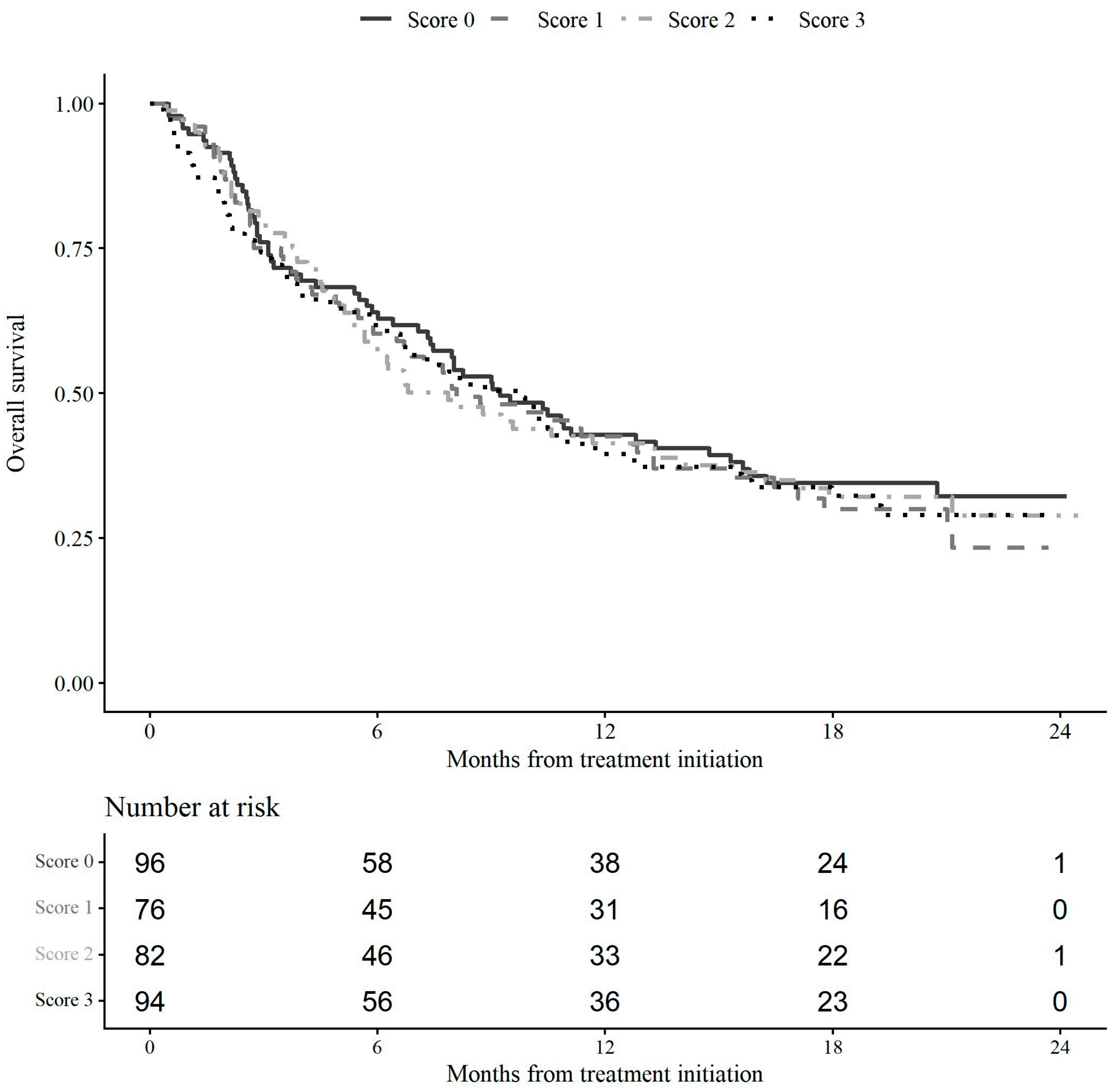

Supplementary Figure S1.
